# Medication administration errors in an intensive care unit in Ethiopia

**DOI:** 10.1186/1755-7682-5-15

**Published:** 2012-05-04

**Authors:** Asrat Agalu, Yemane Ayele, Worku Bedada, Mirkuzie Woldie

**Affiliations:** 1 Department of Pharmacy, Wollo University, College medicine and Health Sciences, P. O. Box 11 45, Dessie, Ethiopia; 2 College of Public Health and Medical Sciences, Jimma University, Jimma, Ethiopia

**Keywords:** Medication error, Medication administration error, Intensive care unit

## Abstract

**Background:**

Medication administration errors in patient care have been shown to be frequent and serious. Such errors are particularly prevalent in highly technical specialties such as the intensive care unit (ICU). In Ethiopia, the prevalence of medication administration errors in the ICU is not studied.

**Objective:**

To assess medication administration errors in the intensive care unit of Jimma University Specialized Hospital (JUSH), Southwest Ethiopia.

**Methods:**

Prospective observation based cross-sectional study was conducted in the ICU of JUSH from February 7 to March 24, 2011. All medication interventions administered by the nurses to all patients admitted to the ICU during the study period were included in the study. Data were collected by directly observing drug administration by the nurses supplemented with review of medication charts. Data was edited, coded and entered in to SPSS for windows version 16.0. Descriptive statistics was used to measure the magnitude and type of the problem under study.

**Results:**

Prevalence of medication administration errors in the ICU of JUSH was 621 (51.8%). Common administration errors were attributed to wrong timing (30.3%), omission due to unavailability (29.0%) and missed doses (18.3%) among others. Errors associated with antibiotics took the lion's share in medication administration errors (36.7%).

**Conclusion:**

Medication errors at the administration phase were highly prevalent in the ICU of Jimma University Specialized Hospital. Supervision to the nurses administering medications by more experienced ICU nurses or other relevant professionals in regular intervals is helpful in ensuring that medication errors don’t occur as frequently as observed in this study.

## Introduction

It has been claimed that drug therapy is the most common intervention prescribed by physicians, and the distribution and administration of medication represents a major duty of hospital-based pharmacists and nurses [1]. A survey report from the United States (2007) showed that 97% of nurses in the country were worried about medication errors, and nearly all (99%) believe that there is a grave risk to patients if errors occurred [2]. Moreover, it was reported that errors associated with medications are the most frequent cause of adverse medical events [3].

Medication errors (MEs) are any preventable events that may cause or lead to inappropriate medication use which can harm patient. Medication errors are major issues in the health care setting and are one of the most common types of medical errors. Medication errors can occur during prescribing; dispensing and administration of medications, and in primary, secondary and tertiary care settings. The underlying cause for such errors may be professional practice, health care products, procedures and systems [4-6].

According to a review article involving 113 intensive care units in 27 countries (2009) medication administration errors were 74.5%. The medication errors are more common in the ICU probably due to poly-pharmacy and the stressful environment. However, majority of the errors were not due to reckless on the part of health care providers, but occurred as a result of the speed and complexity of the medication use cycle, faulty systems, processes and conditions that lead people to make mistakes or fail to prevent them [4,7-11]. Two other observational studies from France (2003) and Switzerland (1998) have also found that medication administration error rates in the acute-care setting varied between 14.9% [12] and 32.4% [13], respectively. Thus, medication errors are frequent, serious and predictable in critical care units. But there is wide variation in the definition and rates of MEs in the ICUs and the methods used to detect them [4,10,11,14-17].

Specific consideration of medication administration also shows that the problem is significant. On their literature review, McBride-Henry and Foureur presented a comprehensive definition of medication administration error given by Wolf (2006), who defined it, as ‘mistakes associated with drugs and intravenous solutions that are made during the prescription, transcription, dispensing, and administration phases of drug preparation and distribution’ [18]. Although the standard is for nurses to check the ‘five rights’ of medication use—right patient, right medication, right dose, right route, right time [19]—only 34% of dispensing and 2% of administration errors are caught prior to reaching the patient in USA (1995 ) [20]. A retrospective analysis of mortalities associated with medication error showed that the most common types of errors resulting in patient death were wrong dose (40.9%), wrong drug (16%), and wrong route of administration (9.5%). Moreover, it was reported that fatal medication errors accounted for approximately 10% of medication errors reported in U.S.A (2001) and were most frequently the result of wrong dosage and administration of a wrong drug [21].

The major consequences of medication errors are patient morbidity and mortality. These can affect patients, families, and health care providers indirectly by cost implications, prolonged hospital stays and psychological impact since errors erode public confidence to health care services [4,10]**.** Although medication errors of all sorts are well investigated throughout much of the developed world, the issue has only rarely been researched in the developing countries including Ethiopia. Consequently, data regarding medication errors in Africa in general and Ethiopia in particular are scarce [22]. Information regarding ME particularly in the ICUs of Ethiopian health institutions is non-existent. This study was hence intended to avail baseline information regarding medication administration errors rates in the ICU of a teaching hospital found in the Southwestern region of Ethiopia.

## Methods

### Study area

This prospective cross-sectional study was conducted as part of a broader study which collected data on medication prescribing and administration errors from February 7 to March, 24, 2011 in the ICU of JUSH [23]. JUSH is a teaching hospital located in Jimma town, in Southwestern Ethiopia, 350 Km from the capital city Addis Ababa. It is the only referral hospital in Southwest Ethiopia with 450 beds and 558 health professionals where a multidisciplinary team of diverse professionals provide a range of health care services for approximately 9000 inpatients and 80,000 outpatients each year. The ICU serves patients who deserve intensive care from all of the clinical departments and has a capacity of 6 beds. Medication distribution is centralized and there is no floor-based decentralized pharmacy service for patients in the ICU [24].

### Subjects

All medication administration interventions to all critically ill patients in the ICU by nurses during the data collection period were included. Data regarding administration of medications were collected by directly observing day time medication administrations and review of medication charts for off-duty medication administrations using pre-tested data collection format.

### Data collection process

Data was collected by trained one BSc. Nurse and two BSc. Pharmacists. The content of the data collection format was designed to record patient demographics and all the data regarding the patients’ medication intervention, dates and times that drug was prescribed including name of the medication, dosage forms, doses given, frequency and duration of medications prescribed. Demographic information about patients was obtained from patient card and medication administration records. Data on medication administration were collected by directly observing all day time (6:00 am to 6:00 pm medication administrations. But on top of the day time observations medication chart review was carried out to collect data on the off duty (7:00 pm till 6:00 am) drug administrations. What was observed and recorded in the medication charts was written down during data collection including all details about the medication.

### Data analysis and determination of medication error rate

Medication administration errors were identified by comparing medication administrations observed/found with what the prescribers ordered. Medication administration error was recorded when discrepancy was found between what was ordered and administered to the patient. Then data were edited, coded and entered into SPSS windows version 16.0. Descriptive statistics was used to measure the magnitude and type of the problem under study. Medication administration rate was calculated as the percentage of errors identified among the total number of medication administrations during the study period.

### Ethical considerations

Prior to the study ethical approval was obtained from the Ethical Review Board of Jimma University. The management of the hospital was requested for cooperation with formal letter. Written consent was obtained from the nurses, physicians and patients included in the study and names of patients and the health professionals were replaced with their initials. All data obtained in the course of the study were kept confidentially and used solely for the purpose of the study.

### Operational definitions

#### Administration errors

implies deviation from the conventional method of administration of a particular drug as ordered by the prescribing physician.

#### Complex regimen

prescription of more than three drugs to one patient at the same time.

#### Antibiotics

in this study are used to mean antibacterial drugs.

## Results

### Characteristics of interventions and respondents

This study included 1200 medication administration interventions to 54 patients in the ICU of JUSH during the study period. Majority of these patients (55.6%) were females and 35 (64.8%) of the patients fall in the age range of 18–50 years with mean age of 33.11 (± 16.78) years. Forty (74.1%) of the patients were admitted to other wards before they were admitted to the ICU. Twenty eight (51.9%) of the patients were unconscious and 38 (70.4%) received complex regimen, with an average of 4.44 (±2.11) different drugs prescribed at the same time. Patients stayed an average of 4.79 (±4.716) days in the ICU till death/transfer to other wards. Average number of co-morbid conditions per patient was 2.32 (±1.59) disease conditions (Table 1).

**Table 1:** Characteristics of patients admitted to the ICU of JUSH, April 2011 (n = 69)

**Characteristics**		**Frequency (%)**
Age	<18 years	9 (16.7)
	18-50 years	35 (64.8)
	>50 years	10 (18.2)
Sex	Male	24 (44.4)
	Female	30 (55.6)
State of patient	Conscious	26 (48.1)
	Unconscious	28 (51.9)
Regimens taken	Complex	38 (70.4)
	Not complex	16(29.6)
State of admission	emergency	14 (25.9)
	From other wards	40 (74.1)
Length of ICU stay	<4 days	25 (46.3)
	> = 4 days	29 (53.7)

Nine nurses (4 BSc. degree and 5 diploma holders) participated in medication administration during the 6 weeks of data collection. Majority of them were in the age group of 20–25 years and were males. Five of them had experience of working in the ICU for more than 12 months and 4 worked for less than 6 months in the ICU (Table 2).

**Table 2:** Characteristics of nurses involved in administration of medications in the ICU of JUSH, April 2011 (n = 9)

**Characteristics**		**Frequency**
Age (years)	20-25	6
	>26	3
Sex	Male	7
	Female	2
Qualification	BSc. Nurse	4
	Diploma	5
Work experience in the ICU	3-6 months	5
	>12 months	4

### Medication errors

**Figure 1:**
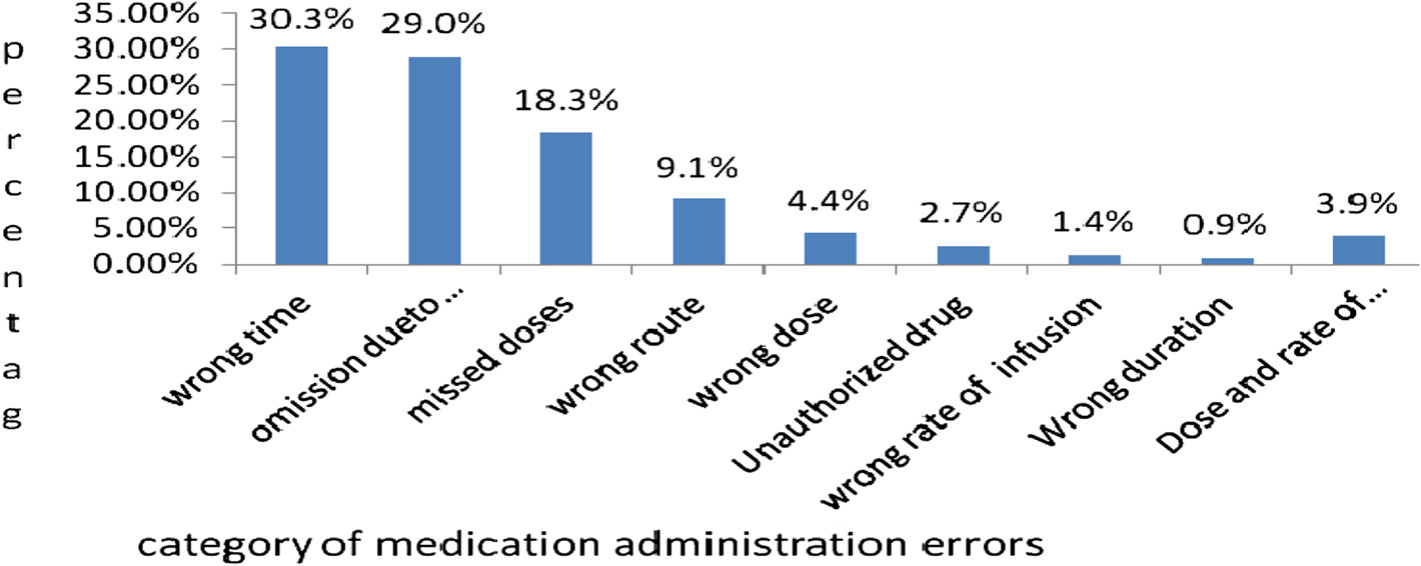
Medication administration error categories in the ICU of JUSH, April 2011.

Out of the 1200 medication administration interventions, more than half (51.8%) were labeled as medication administration errors. Wrong timing, omission due to unavailability and missed doses contributed for 200 (30.3%), 192 (29.0%), 121 (18.3%) of the medication administration errors, respectively. For 26 (3.9%) of the drugs administered, dose and rate of infusion was not determined with appropriate calculations and administration was done arbitrarily (Figure 1).

Drugs associated with medication administration errors were classified according to their therapeutic category. Accordingly, antibiotics (36.7%) and analgesic/antipyretics (13.7%) were the most commonly encountered categories of medications in the medication errors followed by anticonvulsants (10.3%) (Table 3).

**Table 3:** Therapeutic categories of medications associated with medication administration errors in the ICU of JUSH, April 2011

**Drug category**	**Frequency (%)**
Antibiotics	228 (36.7)
Analgesic/antipyretics	85 (13.7)
Anticonvulsants	64 (10.3)
CNS drugs	62 (10.0)
Cardiovascular drugs	61 (9.8)
GI drugs	53 (8.5)
Opioid	26 (4.2)
Others*	42 (6.8)
Total	621 (100)

*Others = Hematologic, anesthetics, anti-parasitic, anticoagulants, anti-thyroids, corticosteroids.

Consideration of specific drugs showed that administration errors were commonest in diclofenac (11.6%), metronidazole (9.5%) and ceftriaxone (9.3%) (Table 4). Moreover, Table 5 below presents some examples of medication administration errors encountered together with what should have been done instead.

**Table 4:** Top ten drugs involved in medication administration error in the ICU of JUSH, April 2011

**Drug category**	**Frequency (%)**
Diclofenac	72 (11.6)
Metronidazole	59 (9.5)
Ceftriaxone	58 (9.3)
Diazepam	51 (8.2)
Cimetidine	48 (7.7)
Phenytoin	45 (7.2)
Crystalline penicillin	27 (4.3)
Ampicillin	24 (3.9)
Pethidine	19 (3.1)
Lovastatin	18 (2.9)
Others*	200 (32.2)
Total	621 (100)

*Others = paracetamol, hydrocortisone, methyldopa, MgSO4.

**Table 5:** Examples **of medication administration errors in the ICU of JUSH, April 2011**

**S. no**	**Examples of medication administration errors**
1	Crystalline penicillin was administered 5 times a day instead of 6 times for all patients, i.e. it wasn’t administered at 2:00 am for all patients
2	Metronidazole IV was mostly missed in the ICU because of lack of the drug in that preparation
3	Quinine IV was mostly missed because it wasn’t available
4	Lovastatin was mostly missed because of lack of the drug
5	Rate of administration of dopamine was 80drops/min which was different from what was labeled on the IV fluid bag, i.e. 30 drops/min for a 60 years old male patient
6	Doses of ceftriaxone were missed for a 35 years old female due to lack of the drug
7	Dose and rate infusion of metronidazole was arbitrarily determined for a 4 years child (250 mg was given in 500 mg/100 ml )
8	Ampicillin was given to an 8 years old male child although the order sheet reads as Cloxacillin
9	An 18 years female patient continued taking methyldopa even after the physician ordered to discontinue the medication
10	Most medications in the morning were being given after 7:00 am instead of 6:00 am
11	Most medications in the afternoon (especially during the weekends) were given before 5:00 pm
12	The morning dose of ceftriaxone was missed for an 8 years female child

## Discussion

According to this study the frequency of medication administration errors was 621(51.8%). Administration errors in current study were higher as compared to an earlier finding reported by a study involving 205 ICUs in 29 countries (46.0%) [14]. Moreover, the rate of administration errors in this study was extremely higher than the findings reported from London (7.0%) [25]. Conversely, a much lower administration error rate was reported in our study as compared to the intensive care and general care units in Pennsylvania, U.S.A (2010) [26]. The difference between the finding of the current study and those of others cited here might be partly explained by the nature of the study settings. However, difference in methods used to calculate medication administration error rate in the studies must be considered on top of lack of trained personnel, appropriate medication and other necessary facilities in the current study area. Irrespective of the reasons, however, medication errors of this magnitude are likely to result in harming the patient and erode public confidence in medical care.

In this study the common medication administration errors were wrong timing (30.3%), omission due to unavailability (29.0%), and missed doses (18.3%). All of these types of errors were higher when compared with those reported from London [25] but lower than the findings in Alabama, Birmingham (2008) [27]. As compared to the findings reported from France, wrong time of administration was higher in this study while the frequency of wrong rate of administration was lower [28]. Improper timing might result in low plasma concentration of medications for those given after the scheduled time interval and increased concentration for those drugs given before recommended time. Either of these consequences is obviously not good to the patient.

On the other hand, in this study the three most common categories of medications associated with administration errors were antibiotics (36.7%), analgesic/antipyretics (13.7%) and anticonvulsants (10.3%). This was not the case in a study involving 113 intensive care units in 27 countries in 2009 where cardiovascular drugs antimicrobial (9.0%), (8.0%), coagulation related (7.0%), diabetic drugs (6.0%) took the leading shares [7]. Unlike a study reported from South Africa in 2006, antibiotics, analgesic/antipyretics and anticonvulsants were highly prone to administration errors in this study [29]. This might be related to the difference in the prescribing pattern, availability and affordability of drugs and time of administration of drugs in the ICU (i.e. those drugs most frequently prescribed and administered might be related with higher levels of administration errors). Such level of error associated with administering antibiotics is a concern as this might result in drug resistance and adverse drug reactions, treatment failure and death in the worst scenario.

According to the present study diclofenac (11.6%), metronidazole (9.5%), ceftriaxone (9.3%) and diazepam (8.2%) were the four common specific drugs prone for administration errors. A similar study involving 113 intensive care units in 27 countries in 2009 reported that vasopressors and catecholamine (8%), insulin (6%), coagulation related (7%), electrolytes (6%) were associated with administration errors [7]. This difference might be due to difference in drugs available in the study hospitals and pattern of prescribing which in turn is related with the prevalence and incidence of cases in the study areas.

The findings of this study must be interpreted keeping in mind the fact that we did not assess severity of errors and outcome of treatment. Errors were rated by accounting one error for one dose administered i.e. at least one error per dose given. Some information (including exact dose, time, route of medications administered) regarding medications administered were missing in the medication charts. Errors during preparation, delay in administration of medication since ordered, and dosage forms were not included in determining administration errors. Furthermore, although we claimed that nurses were unaware about the purpose the study, they might have some sense of it particularly towards the end of the study period. This could have influenced their practice during the study period resulting in social desirability bias.

In Conclusions, medication errors at the administration phase were highly prevalent in the ICU of the JUSH. Antibiotics were most commonly encountered drug categories in administration errors in the ICU. With the increasing complexity of care in critically ill patients, organizational factors such as error reporting systems and routine checks could possibly help in handling the problem of medication error. Hospital managers should strive to create better awareness about the possibility of medication errors and its aftermath among health care professionals. Moreover, the nurses administering medications need to be supervised by more experienced ICU nurses or other relevant professionals in regular intervals to ensure that medication administration errors don’t occur as frequently as observed in this study. It is also recommended that the hospital authorities insure procurement and availability of appropriate medications to the ICU. These will definitely contribute to the prevention of medication administration errors which could likely be dangerous to the lives of the already critically ill patients. This in turn will build public confidence in the care provided at the health facility.

## Competing interests

The authors declare that they have no competing interests.

## Authors’ contributions

AA was involved in the design of the study, data analysis, and interpretation of the findings, report writing and manuscript preparation. YA was involved in the design of the study, data analysis and review of report. WB was involved in the design of the study, analysis and interpretation of the data, and review of the report. MW was involved in the design of the study, analysis and interpretation of the data, and writing and review of the report and manuscript. All authors read and approved the final manuscript.
